# Supplementary material for: Population Dynamics of Intestinal Enterococcus Modulate Galleria mellonella Metamorphosis
Source: Microbiol Spectr. 2023 Jun 26;11(4):e02780-22. doi: 10.1128/spectrum.02780-22 (PMC10434003; doi:10.1128/spectrum.02780-22)

SUPPLEMENTAL MATERIALS

Population dynamics of intestinal *Enterococcus* modulate *Galleria mellonella* metamorphosis

Hyun Gi Kong[1,3], Jin-Soo Son[1], Joon-Hui Chung[1], Soohyun Lee[1], Jun-Seob Kim[1,2*], and Choong-Min Ryu[1*]

[1]Infection Disease Research Center, KRIBB, Daejeon 34141, South Korea

[2]Department of Nano-Bioengineering, Incheon National University, Incheon 22012, South Korea.

[3]Department of Plant Medicine, Chungbuk National University, Cheongju, 28644, South Korea.

***Co-correspondence**

***For correspondence. E-mail** junkim@inu.ac.kr; cmryu@kribb.re.kr

Running title: Gut bacteria modulate insect metamorphosis

Keywords: Microbiota alteration, Insect metamorphosis, Host–microbe interactions

SUPPLEMENTARY TABLES

Table S1. Primer Sequence details

Primer name	Sequence 5´-3´	Reference
Gallerimycin F	GAAGTCTACAGAATCACACGA	Lange *et al.* (2018)
Gallerimycin R	ATCGAAGACATTGACATCCA	
Apolipophorin III F	AGACTTGCACGCCATCAAGA	Lange *et al.* (2018)
Apolipophorin III R	TGCATGCTGTTTGTCACTGC	
Gloverin F	GTGTTGAGCCCGTATGGGAA	Lange *et al.* (2018)
Gloverin R	CCGTGCATCTGCTTGCTAAC	
Cecropin-D F	CTGTTCGTGTTCGCTTGTGT	Lange *et al.* (2018)
Cecropin-D R	GTAGCTGCTTCGCCTACCAC	
IMPI-F	TAGTAAGCAGTAGCATAGTCC	Dubovskiy *et al.* (2016)
IMPI-R	GCCATCTTCACAGTAGCA	
β-Actin f	CCCTGTGCTGCTCACCGA	Lange *et al.* (2018)
β-Actin r	ACAGTGTGGGTGACCCCGTC	

SUPPLEMENTARY FIGURES LEGENDS

Fig S1. Heatmap of gene expression of *G. mellonella* according to metamorphosis. Normalized counts obtained with DESeq transformed to hellinger distance were used to generate heatmaps showing overexpressed (red) and underexpressed (blue) genes for two developmental conditions (larva and pupae).

Fig S2. Sparse Canonical Correlation analysis. Sparse canonical correlation analysis was performed to analyze the correlation between microbiome data and RNAseq expression in the larva and pupae stages of Galleria mellonella. The database was performed with taxon and gene lists that differ by more than 2-fold change, and analyzed using the iSFun package of the R program Gene ontology analysis was performed on the genetic data from the obtained data using Panther, and it was created as a heatmap through R program.

Fig S3. Hemolymph protein profile analysis. In-gel chymotryptic digestion for each protein segment < 25 kDa on electrophoresis was analyzed with LC-ESI-Q-TOF-MS for protein profile analysis. The fasta sequence DB of "Ditrysia and antimicrobial" (including 20 Galleria antimicrobial protein sequences) and the fasta sequence DB of "Lepidoptera" (including 17,496 Galleria sequence DB) are used as databases.

Fig S4. Confirmation of the presence of Enterococcus spp in the intestine after vancomycin treatment. *E. innesii* (Eg^{refR}) and *E. mundtii* (Em^{refR}) marked with rifampicin antibiotics were used to confirm the removal of Gram-positive bacteria from the intestine after vancomycin treatment. After feeding each bacteria, the reduction of bacteria by antibiotic treatment was tested by culturing in rifampicin-TSA.

References

Dubovskiy IM., Grizanova EV., Whitten MMA., Mukherjee K., Greig C., Alikina T., et al. (2016). Immuno-physiological adaptations confer wax moth *Galleria mellonella* resistance to *Bacillus thuringiensis*. Virulence 7, 860–870.

Lange A., Schäfer A., Bender A., Steimle A., Beier S., Parusel R., et al. (2018). *Galleria mellonella*: A novel invertebrate model to distinguish intestinal symbionts from pathobionts. Front. Immunol. 9, 2114.

Supplementary Figure 1

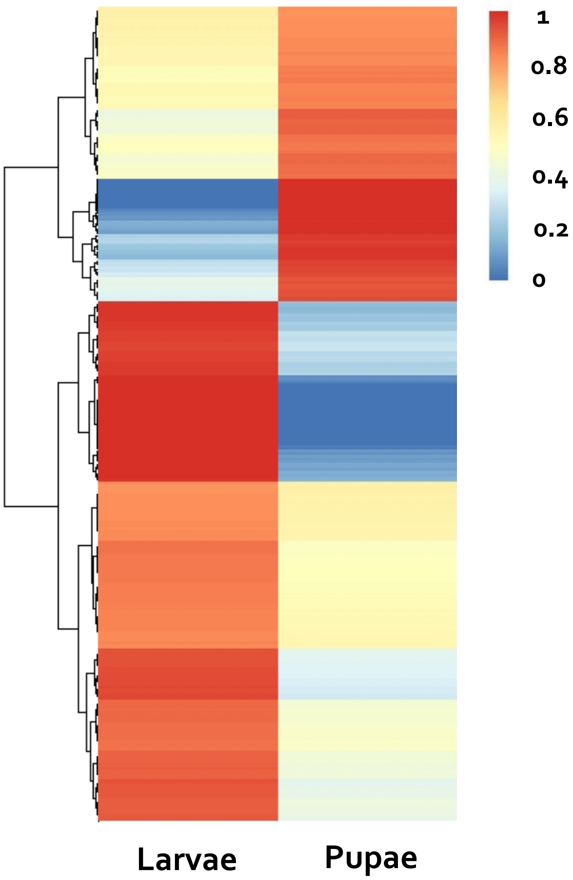

Supplementary Figure 2

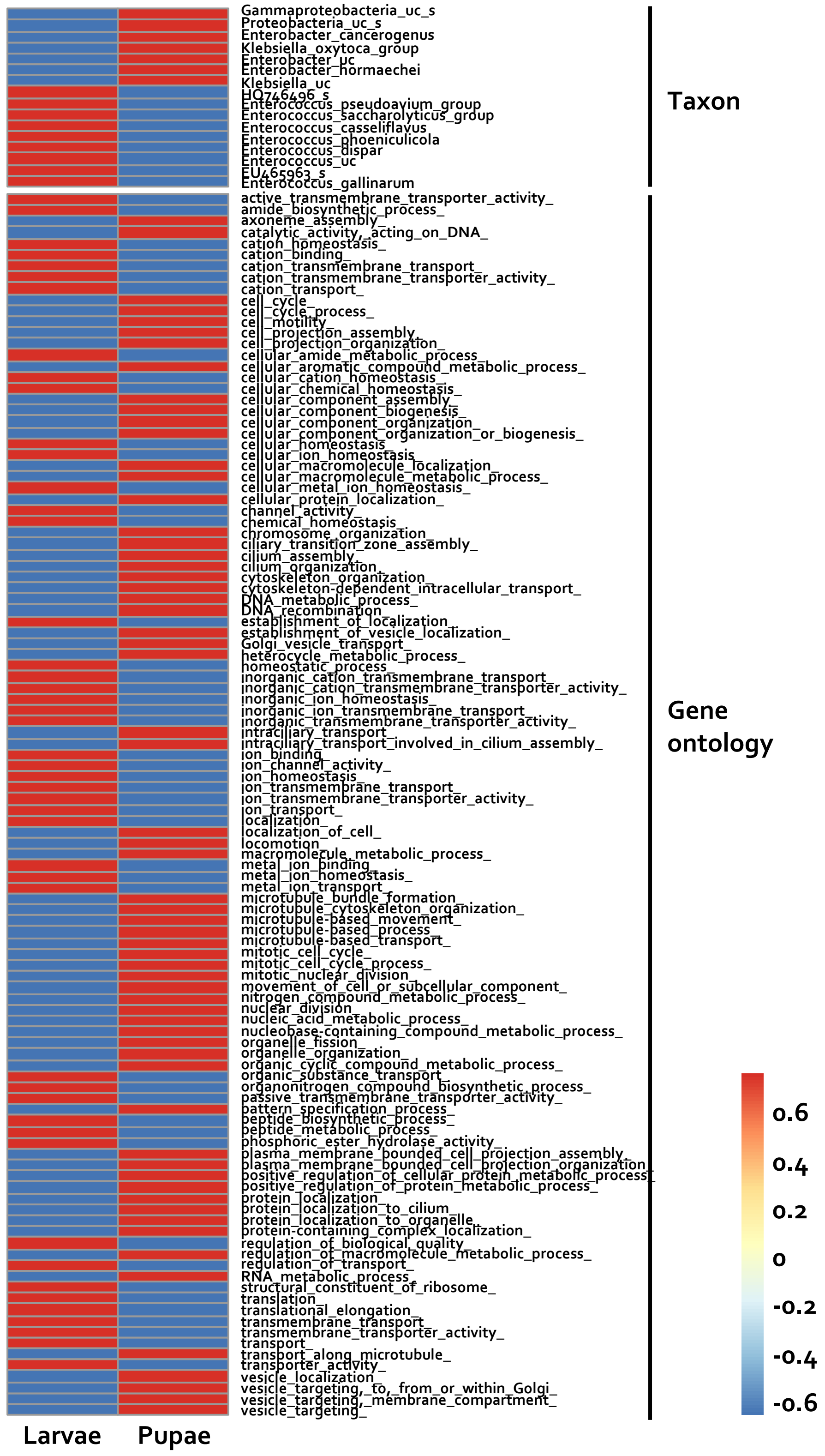

Supplementary Figure 3

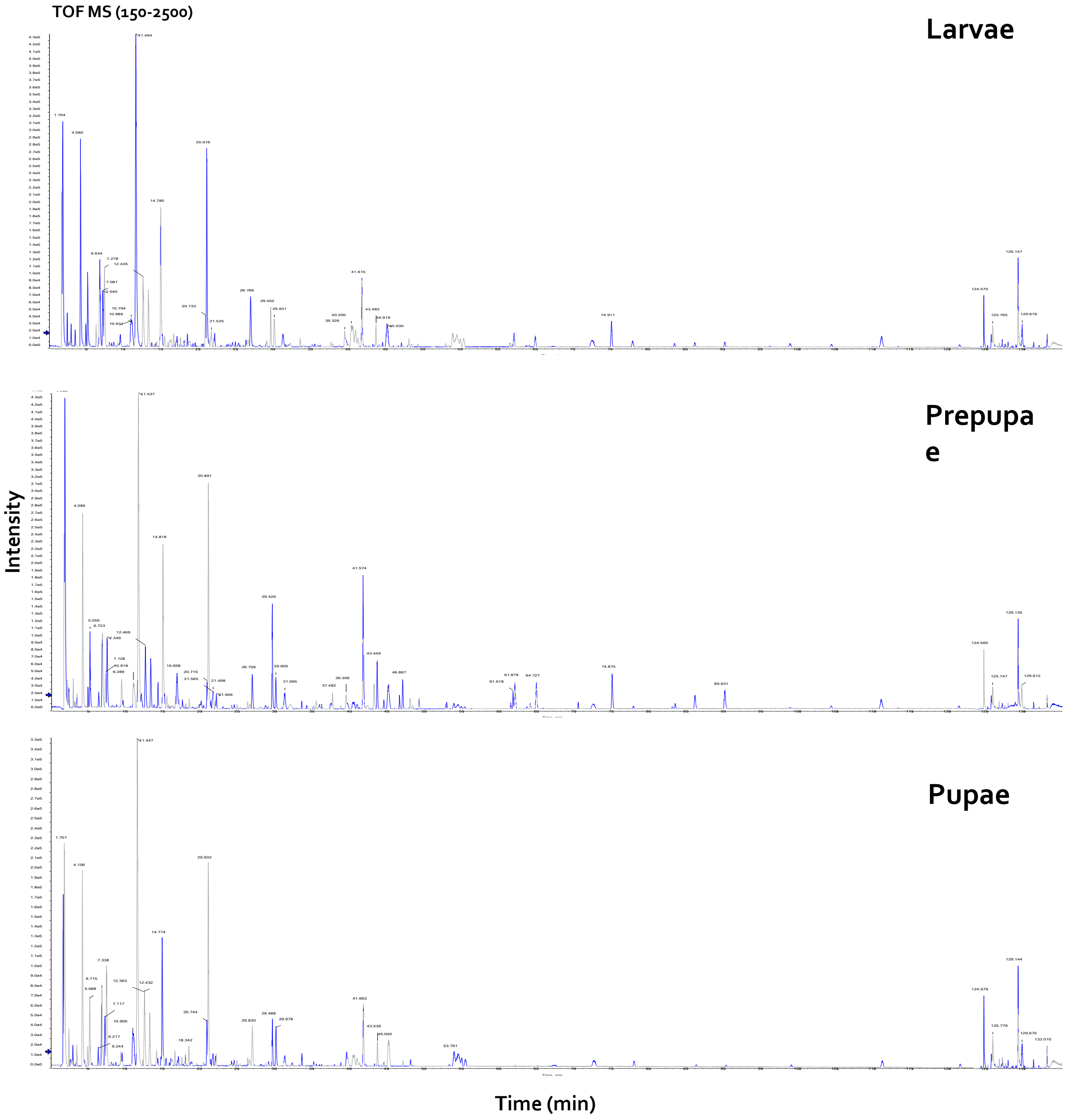

Supplementary Figure 4

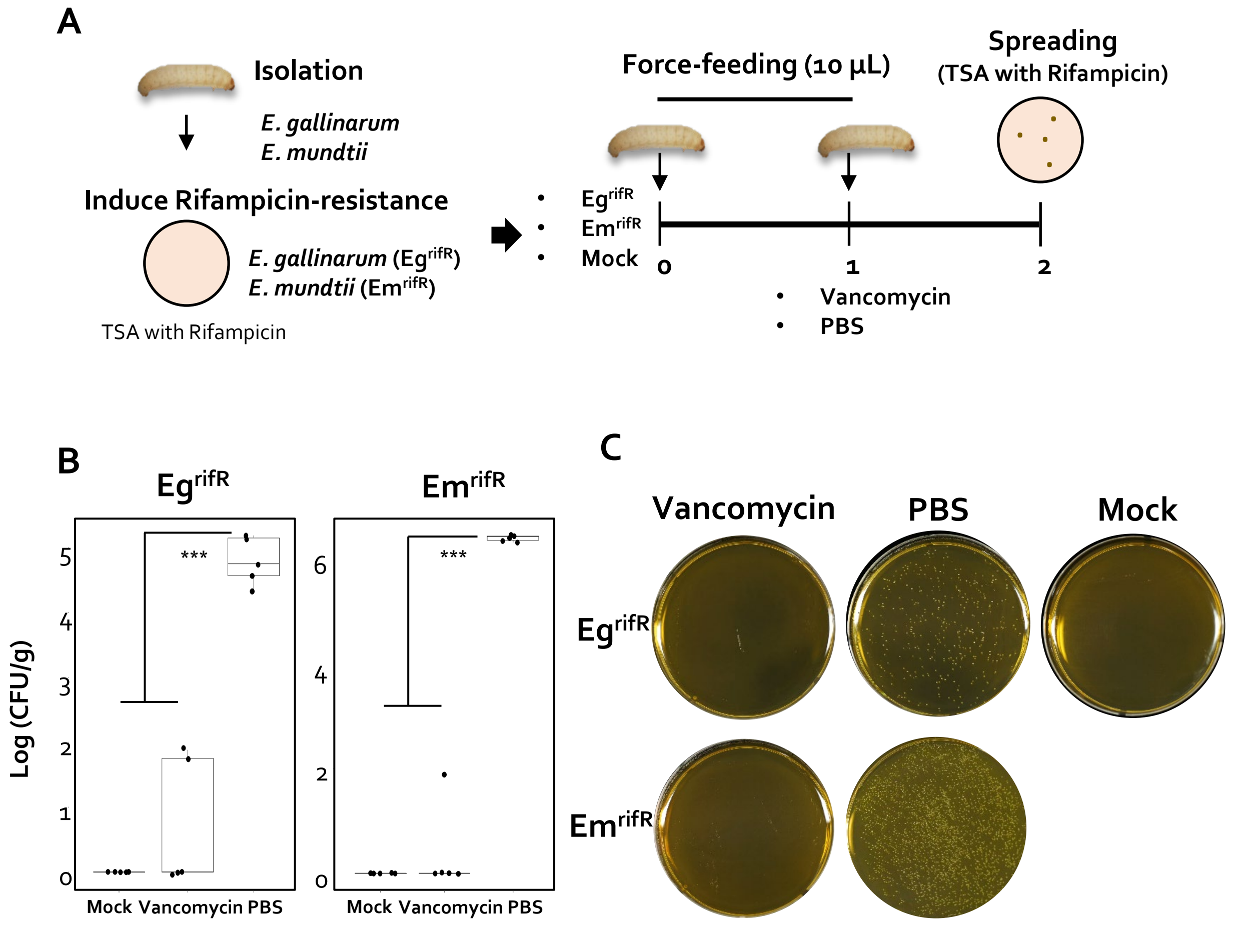

Supplement: Supplemental file 1 — Supplemental material. Download spectrum.02780-22-s0001.pdf, PDF file, 1.5 MB [file spectrum.02780-22-s0001.pdf]
